# Prediction as a basis for skilled reading: insights from modern language models

**DOI:** 10.1098/rsos.211837

**Published:** 2022-06-15

**Authors:** Benedetta Cevoli, Chris Watkins, Kathleen Rastle

**Affiliations:** ^1^ Department of Psychology, Royal Holloway, University of London, Egham, UK; ^2^ Department of Computer Science, Royal Holloway, University of London, Egham, UK

**Keywords:** prediction, eye movements, GPT-2, language models, reading

## Abstract

Reading is not an inborn human capability, and yet, English-speaking adults read with impressive speed. This study considered how predictions of upcoming words impact on this skilled behaviour. We used a powerful language model (GPT-2) to derive predictions of upcoming words in text passages. These predictions were highly accurate and showed a tight relationship to fine-grained aspects of eye-movement behaviour when adults read those same passages, including whether to skip the next word and how long to spend on it. Strong predictions that were incorrect resulted in a prediction error cost on fixation durations. Our findings suggest that predictions for upcoming words can be made based on the analysis of text statistics and that these predictions guide how our eyes interrogate text at very short timescales. These findings open new perspectives on reading and language comprehension and illustrate the capability of modern language models to inform understanding of human language processing.

## 1. Introduction

Reading is not an inborn human capability, and yet, literate English-speaking adults read at nearly 250 words per minute [1]. There has been substantial interest in understanding the extent to which *prediction* of upcoming words underpins this skilled language behaviour. Eye-tracking (e.g. [2]) and electrophysiological studies (e.g. [3]) have established that words that are highly predictable given the preceding context are easier to recognize than those that are less predictable (see [4] for a review). To illustrate, research suggests that the word *vegetarian* is easier to recognize in (a) than in (b) below (adapted from [2]).

(a) *Megan decided to stop eating meat. She declared herself a vegetarian today*.
(b) *Megan went to lunch with colleagues. She declared herself a vegetarian today*.

However, despite decades of research investigating predictability effects in reading, there is considerable disagreement about whether readers routinely predict upcoming words. Our study offers a new perspective on this debate by using a modern language model to delineate the impact of predictions on eye-movement behaviour across the time course of natural reading.

Several lines of argument suggest that skilled readers do not routinely predict or anticipate upcoming words (see [5] for discussion). One set of arguments suggests that the predictability effects observed in the literature reflect *integration* processes rather than prediction. In the example above, the word *vegetarian* would be recognized more quickly in (a) than in (b) not because the reader has anticipated that word in advance of encountering it but because it is easier to integrate in the unfolding context (see [6,7] for discussion). However, while prediction is difficult to disentangle from integration, the fact that predictability influences very early eye-movement measures including the probability of skipping a word (e.g. [2]) seems unlikely to be consistent with a full integration account [5]. Yet, even if there is some role for prediction, others have argued that natural language is too unconstrained to make prediction a viable processing strategy in most contexts [8]. Finally, while there is relative consensus that reading may involve pre-activation of general semantic or morpho-syntactic features (e.g. [9–11]), evidence that readers predict specific, upcoming words is far more limited.

If readers engage in prediction of specific upcoming words, then there should be some evidence of a processing cost when those predictions do not materialize [5]. The strongest demonstration of such a processing cost was reported by DeLong *et al*. [12] in an ERP study. They observed a higher N400 amplitude when readers encountered *articles* that were inconsistent with a strongly predicted target as in the sentence ‘*The day was breezy so the boy went outside to fly an …*’. This finding is important because it implicates pre-activation of a *specific phonological form* (see also [13]). However, this result was not replicated in a pre-registered, highly powered study [14]. Evidence for a processing cost because of inaccurate predictions has also been sought using eye-tracking methodology. Frisson *et al*. [15] monitored fixation duration on unpredictable target words embedded in highly constraining or neutral sentence contexts, as in the below examples (target in bold).

(a) *The young nervous paratrooper jumped out of the **chair** when he heard the shots*.
(b) *The tired movie maker was sleeping in the **chair** when he was woken up by a scream*.

They reasoned that if readers generate specific predictions in highly constraining contexts such as sentence (a) above (in which the word *plane* should be predicted), then there should be some evidence of a prediction error cost on fixations to unpredictable targets, relative to neutral contexts (b). Despite observing the usual predictability effect, no evidence for a prediction error cost was observed on any measure.

Drawing inferences regarding the use of prediction in reading has been hampered by a variety of methodological challenges. In addition to the challenge of disentangling prediction from integration, constraints on stimuli and stimulus presentation in different paradigms make it difficult to determine whether prediction is a phenomenon associated with natural text reading, as opposed to more artificial laboratory paradigms. In electrophysiology studies, words are typically presented one at a time with a fixed duration [16–18]. This unnatural presentation disrupts typical characteristics of natural reading including fixation durations, skips, and regressions. It also elicits predictive processes that may differ from those that occur in normal reading [19–22]. Eye-tracking studies avoid these pitfalls but investigate predictability effects on single targets contained in artificial sentences deliberately designed to be predictable or unpredictable (see [5] for review).

It is also difficult to quantify the predictability of words in different contexts. Most studies in this domain measure predictability as cloze probability (e.g. [2,23,24]). In cloze tasks, participants are presented with a sentence frame and asked to guess the next word. The distribution of participants’ top predictions is then taken to reflect the probability distribution of the target in context. The underlying assumption is that participants' responses reflect their individual probability distributions for upcoming words; however, this assumption has been challenged [25,26]. Most critically, cloze measures underestimate the probability of unpredictable words; such words are rarely indicated by participants and are therefore associated with a cloze probability of zero. The bias against responding with an unpredictable word may hide important variation in the lower range. These limitations are partly mitigated by use of corpus measures of predictability, which use *n*-gram models to compute the conditional probability of a word given a preceding local context [27,28]. This approach allows more accurate estimation of the lower range of predictability; however, these estimates are usually based on local contexts of only a few words and thus ignore background information and long-range dependencies.

The present study advances the debate by using a newly developed language model to characterize the predictability of words in a large eye-tracking corpus of natural passage reading [29] and then relating those metrics to reading behaviour. The language model used is GPT-2, a deep neural network based on a transformer architecture that uses attention mechanisms to focus selectively on words in the text that are most relevant in a context [30]. The model is trained on vast corpora to predict upcoming words accurately given the preceding context. The attention mechanisms allow for training of context-specific word representations (i.e. *contextualized embeddings*). This means that unlike predecessor language models such as LSA [31] or word2vec [32], the learned high-dimensional representation of a single word will vary as a function of its context; for example, representation of the word *bark* will differ if it is in the context of a tree or a dog [33].

In essence, GPT-2 provides a computational instantiation of a next-word prediction strategy that does not rely on deep understanding. The use of GPT-2, therefore, offers opportunity to assess the extent to which prediction is *feasible* in natural reading, and it also provides a means of quantifying precisely the probability distribution of any word in any context, including those words with very low probability. By presenting each word in the eye-tracking corpus sequentially to the model, we are able to extract novel measures that quantify (a) uncertainty *before* a target is encountered and (b) surprisal *when* a target is encountered. This distinction is critically important because it allows us definitively to separate prediction from integration. Our analyses sought to determine whether there is unambiguous evidence that specific predictions made before a target is encountered drive fixations to that target and whether there is evidence that inaccurate predictions yield a prediction error cost.

## 2. Method

The study derived fine-grained predictability metrics from GPT-2 and applied these to eye-movement data derived from the Provo Corpus [29]. The materials, data and analysis scripts for this project can be found at https://osf.io/ypeb2/.

### 2.1. Provo Corpus

This study exploited the Provo Corpus [29], a large and publicly available corpus of eye movements in reading. The corpus consists of eye-tracking data from 84 adult American English native speakers reading 55 short passages derived from a variety of sources, including online news articles, science magazines and fiction. The eye-movement measures of interest to us include whether the target was fixated in the first pass (i.e. before the eyes move rightward from the target), first fixation duration (length of the first fixation on a word), gaze duration (sum of all fixation durations in the first pass), total reading time (sum of all fixation durations), and regressions back to the target. The corpus also includes predictability norms derived from an online word-by-word cloze task completed by 470 native American English speakers. These norms provide information about the percentage of participants that accurately predicted the target as the next word.

### 2.2. Language model

Predictability metrics were derived from a pre-trained version of GPT-2 [30] (model version: *base*; 12-layer, 768-hidden, 12-heads, 117 M parameters) with no further fine-tuning. This version of the model was trained for optimal probabilistic prediction according to the minimal cross-entropy criterion and was accessed through the Hugging Face Transformers library [34]. We used the python scientific stack to compute predictability metrics from the model.

**Figure 1.**
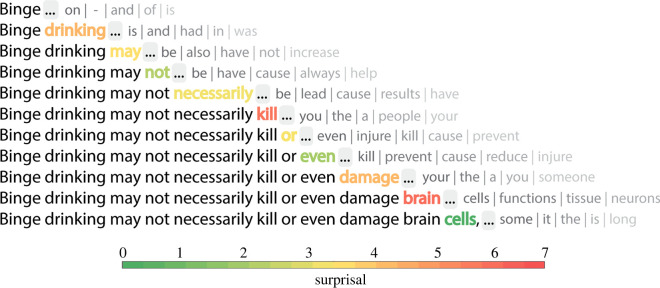
Illustration of language model next-word predictions (on the right in grey) given the preceding context (on the left in black or coloured text). The transparency of the grey predictions indicates their strength. The colour of the final word indicates the language model surprisal when the target word is encountered.

The language model's predictions about an upcoming word in a passage are expressed in terms of the likelihood of each word in the model's vocabulary occurring as the next word. These predictions consist of a vector of logit prediction scores for each vocabulary token, hereafter referred to as the ‘prediction-probability vector’. We derived likelihood values by normalizing these scores with a softmax function. An incremental moving window was used to calculate predictability information for each word in the corpus passages (for a total of 2685 tokens and 1191 types). The language model's predictions were analysed for each word given the preceding context. Starting from the very beginning of a text passage, the first word is provided to the model and predictability information about the subsequent word (the *target*) is extracted. Then, the first and the second word are provided to examine predictions of the third word (the new target), and so on (figure 1). In this way, predictability information based on the language model's predictions were computed for each of the 2685 words in the corpus.

We derived two metrics from the prediction-probability vectors. The first metric quantified the uncertainty of the context preceding a target in terms of the *entropy* of the prediction-probability vector. This metric reflects the extent to which a context is neutral or constraining in nature. Low entropy values are typical of constraining contexts as only a few words have a very high probability of occurring next (e.g. *The elephant drank using its long* ____). By contrast, neutral contexts are represented by high entropy values as many words are equally likely to follow (e.g. *The man looked for the* _____). The second metric quantified the predictability of a target word given the preceding context as *surprisal*, the negative natural log of the language model's probability of that word. It is important to stress that in the computation of a context's entropy, no information about the succeeding target word is provided to the language model. Entropy reflects the state of the language model's predictions *before* the target word is encountered, while surprisal reflects the state *when the target word is encountered*. Thus, while an effect of surprisal on eye-movement behaviour might be ascribed to prediction or integration processes, an effect of entropy *must* be ascribed to prediction [6]. Though the concepts of entropy and surprisal are related, they can be distinguished in natural language (table 1 for examples), and we monitored our statistical models closely for excessive collinearity (see Results).

**Table 1.** Examples from passages in the Provo corpus with varying entropy and surprisal metrics as derived from the language model's predictions.

entropy	surprisal	context	predictions	target
high	high	all that the	*world government media*	brain
low	high	binge drinking may not necessarily kill or even damage	*your the a*	brain
high	low	…but it can block key receptors in the	*brain hippocampus body*	brain
low	low	…but it can block key receptors in the brain and trigger production of a steroid that interferes with	*the brain normal*	brain

If readers predict specific upcoming words, then we would expect to observe a *prediction error cost* when these predictions are inaccurate [5]. Thus, the impact of target surprisal on fixation behaviour should vary as a function of entropy; surprising targets should be particularly detrimental when entropy is low. To examine whether the semantic relationship between the specific prediction and the target modulates any prediction error cost, we computed a final metric for each word in the corpus referred to as *target-prediction similarity*. Target-prediction similarity was operationalized as the cosine similarity between embeddings of the top prediction in the language model and the target. It is important to remember that these are *contextual embeddings* (i.e. representations of a word in a particular context).

## 3. Results

Eye-tracking data were analysed using (generalized) linear mixed-effects models (lme4 package by [35]). In the analysis, reading time measures were log-transformed, as indicated by a Box–Cox test (from the R package car [36]). For visualization purposes, these were back-transformed by exponentiation. In addition to the fixed factors of interest, models included word frequency estimates from SUBTLEX [37], word length in letters, and the number of the word in a sentence as fixed factors as well as random intercepts for participants, items and text passages. The significance of the fixed effects was determined using the Anova function in the car package (type III model comparisons [36]). The models reported below all had a variance inflation factor below 2, suggesting that they do not suffer from excessive collinearity [38]. Full results of all models are available on the OSF site for this project: https://osf.io/ypeb2/.

### 3.1. Validity of the language model predictions

**Figure 2.**
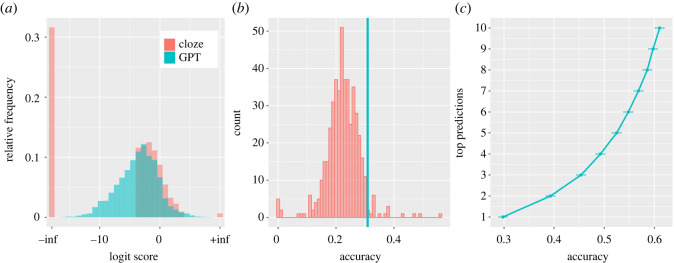
(*a*) The target probability distribution in logits derived from the language model and cloze task (where logit scores of −inf correspond to probabilities of zero and +inf to probabilities of 1). (*b*) A frequency distribution of next-word prediction accuracy for the 470 human participants who contributed cloze data (red) and the language model (blue). (*c*) The accuracy of the language model in predicting the next word based on top *k* predictions, for *k* from 1 to 10 (error bars indicate the standard error of the mean).

Before analysing the influence of the language model predictions on eye movements during natural reading, we examined the validity of these metrics against cloze data for each word reported in the Provo Corpus [29]. The distribution of target probability derived from the cloze task and the language model are compared in figure 2*a*. This figure plots target probability using logit scores as these are more normally distributed and allow superior comparison of the two distributions in the low range. The probability values from the cloze task reflect an average across participants, while the values from the language model reflect the probability of that target occurring relative to every other word in the language model's vocabulary. It is immediately apparent that the probability distributions of the language model and the cloze task are very similar in the upper range but completely diverge in the lower range of the distribution. Whereas the language model provides a continuous distribution of probabilities, the cloze task estimates 32% of the entire dataset to have a target probability of zero. This analysis suggests that the cloze task dramatically underestimates low probability values when compared to the language model.

We then compared the performance of the language model in predicting the next word (given a preceding context) to human performance in doing the same task. Human performance was based on 470 participants each completing the word-by-word cloze task for 5 passages (an average of 227 words each) [29]. On average, participants had an accuracy of 22% (standard deviation 6%; maximum accuracy 56%), whereas the top prediction of the language model was accurate 31% of the time (figure 2*b*). This figure shows that the language model was more accurate in its predictions than most but not all human participants. Figure 2*c* shows that when we increase the prediction window from the top prediction to the top 10 predictions, the accuracy of the language model increases to 61% accuracy.

These assessments of GPT-2 performance advance understanding in several ways. They reveal that the cloze task is insensitive to probabilities at the lower end of the distribution (see also [26]), suggesting that cloze data are not appropriate for investigating prediction in natural reading contexts, in which low probability sequences are frequently encountered. Conversely, these data reveal that the language model not only produces a graded distribution of probability values but also is superior in its predictions to almost all individual participants. Finally, the fact that the language model was able to predict the next word 31% of the time in a natural text corpus, and that this figure rose to over 60% when the model's top 10 predictions were considered, suggests that natural language may be more predictable than has previously been asserted [8].

### 3.2. Eye movements analysis

Having established the language model as a valid source of predictions, we then sought to determine how internal model predictions are related to fine-grained eye-movement behaviour. Thus, we examined the influence of the language model's state (a) *before* participants reach a target (entropy) and (b) *when* participants reach a target (surprisal). We also analysed whether there is evidence that eye-movement behaviour is influenced by the semantic relationship between the predicted and observed target (target-prediction similarity). We investigated the effects of these metrics as appropriate on skipping, reading times and regression probability. Eye-tracking measures of reading times included first fixation duration (length of the first fixation on a word), gaze duration (sum of all fixation durations in the first pass) and total reading time (sum of all fixation durations).

#### 3.2.1. Skipping behaviour

It is well known that not all words are fixated directly and that the probability of skipping a word is modulated by factors including its length, frequency and cloze probability (see [39] for discussion). Words are more likely to be skipped when they are short, high frequency, and predictable from the preceding context. One influential model of eye-movement behaviour suggests that the decision to skip an upcoming word may be based on analysis of that word in the parafovea [40], hence the impact of these target properties on skips.

Our aim was to determine whether entropy influences the probability of skipping an upcoming word over and above any information that might be accessed about that word parafoveally. We, therefore, tested the influence of entropy on whether an upcoming target is skipped but included in the model factors pertaining to the target including length, frequency and surprisal. We specifically tested for an interaction between entropy and surprisal to determine whether any effect of entropy is modulated by whether the prediction derived from preceding context is accurate. We used the following model formula: *SkippingProbability ∼ Entropy × Surprisal + Freq + Length + WordNumberInSentence + (1|ParticipantID) + (1|WordID) + (1|TextID*). Descriptive statistics indicate that 113 690 words were skipped out of the 249 984 observations in the Provo Corpus [29].

**Figure 3.**
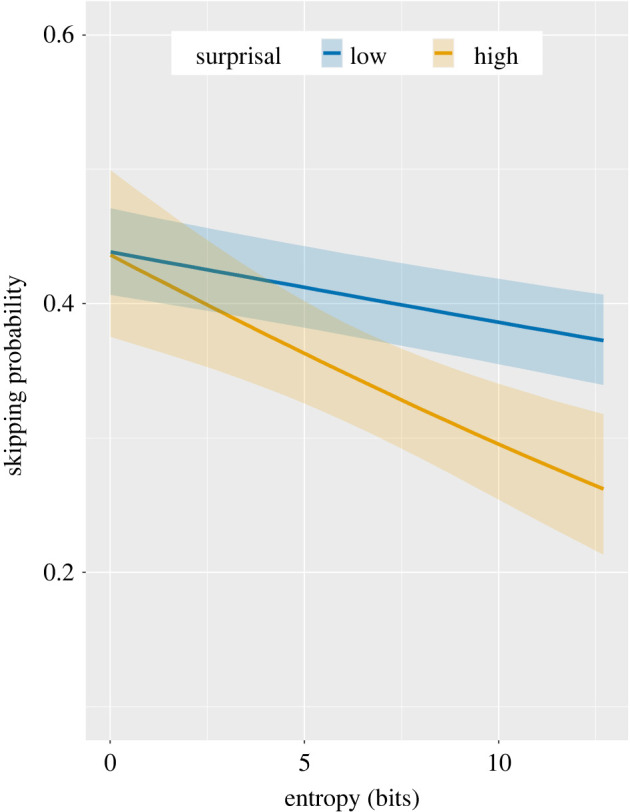
Model estimates of the interaction between entropy and surprisal, expressed in information units (bits), on human skipping probability. Surprisal was split into high and low values for visualization purposes only with equal frequency binning (to create equally distributed categories).

Results revealed that the entropy of the preceding context influenced the probability that a target would be skipped ($\chi^{2}=75.95$, OR=0.93, s.e.=0.01, Z=−8.95, p<0.001). Readers are more likely to skip the upcoming word if the preceding context has lower prediction-probability entropy (more constraining contexts) than higher prediction-probability entropy (more neutral contexts). However, this analysis also revealed a significant interaction between entropy and surprisal on skipping probability ($\chi^{2}=4.25$, *OR* = 0.99, s.e.=0.01, Z=−2.06, p=0.04; figure 3). This result indicates that target surprisal has an impact on skipping probability but only when readers are more uncertain of the next word (higher entropy). In lower uncertainty contexts (lower entropy), the reader acts on the strength of their prediction without regard to whether that prediction is accurate.^1^ Critically, none of these effects can be ascribed to frequency, length or position in the sentence as these variables are included in the model.

#### 3.2.2. Fixation durations

For those targets that were not skipped (136 294 of the original 249 984 observations), we investigated how reading times were influenced by uncertainty of the preceding context (entropy) and target surprisal. We used the following model formula: *DV ∼ Surprisal × Entropy + Freq + Length + WordNumberInSentence + (1|ParticipantID) + (1|WordID) + (1|TextID)*.

**Figure 4.**
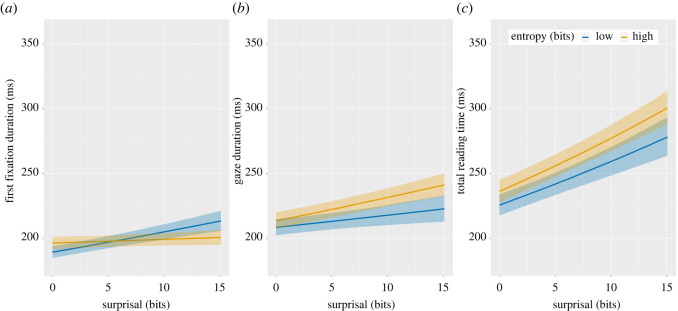
Model estimates of the effect of surprisal and entropy of the preceding context, expressed in information units (bits), on human reading times. Entropy was split into high and low values for visualization purposes only with equal frequency binning. First fixations show a significant interaction between surprisal and entropy (*a*) with surprisal impacting fixations more strongly when entropy is low. The effects of surprisal and entropy are additive on later measures of gaze duration (*b*) and total reading time (*c*).

Results on first fixations (duration of the first fixation on a word) revealed an interaction between surprisal and entropy (β<0.01, s.e.<0.01,  t=−3.86, p<0.001). High surprisal targets lengthened first fixations most strongly when there was high certainty that the target should be something else (low entropy; figure 4*a*). This result is crucial because it reveals a *prediction error cost* in natural reading, a processing penalty when a strong prediction is inaccurate.

Results on later measures revealed significant effects of entropy (β=0.01, s.e.<0.01, t=5.17, p<0.001) and surprisal (β=0.02, s.e.<0.01, t=9.00, p<0.001) on gaze duration (sum of fixations in the first pass). Likewise, there were significant effects of entropy (β=0.01, s.e.<0.01, t=6.16, p<0.001) and surprisal (β=0.05, s.e.<0.01, t=17.82, p<0.001) on total reading time (sum of all fixations; figure 4). In both cases, fixations were longer when there was greater uncertainty in the preceding context, and when targets were more surprising. There was no interaction between these factors on gaze duration (β=0.01, s.e.<0.01, t=1.72, p=0.08) or total reading time (β=0.01, s.e.<0.01, t=0.86, p=0.39).

#### 3.2.3. Regressions

**Figure 5.**
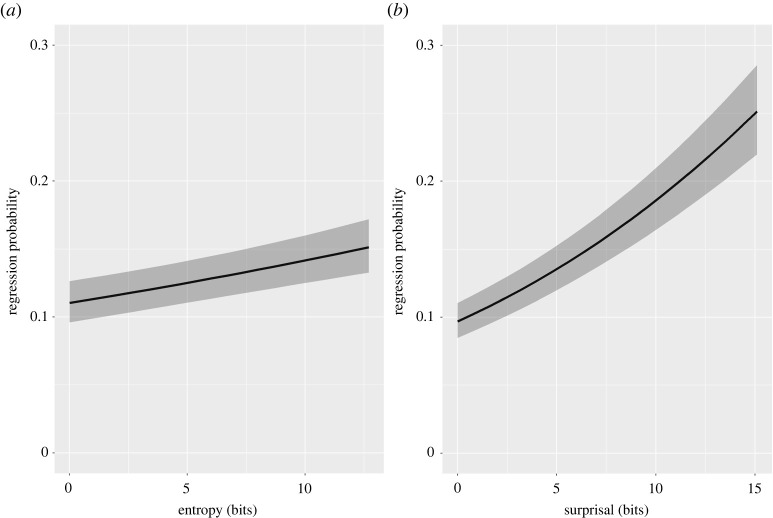
Model estimates of the effect of entropy (*a*) and surprisal (*b*) on human regression probability. Entropy and surprisal are expressed in information units (bits).

We examined the influence of entropy and surprisal on the probability of regressing back to the target using the following model formula: *RegressionProbability ∼ Surprisal × Entropy + Freq + Length + WordNumberInSentence + (1|ParticipantID) + (1|WordID) + (1|TextID)*. Results revealed that readers were more likely to regress back to words that occur in higher entropy contexts (OR=1.08, s.e.<0.01, z=5.83, p<0.001) and that are more surprising (OR=1.25, s.e.=0.02, z=15.09, p<0.001; figure 5). These data suggest that the ramping up of total reading times in low entropy and high surprisal contexts (figure 4*c*) is likely due to regressions back to targets. There was no interaction between entropy and surprisal on regression probability (OR=0.99, s.e.=0.01, z=−1.04, p=0.29).

These findings advance understanding by showing unambiguously that predictions *prior* to encountering a target drive aspects of fixation behaviour at very short timescales. They also provide new insight into the nature of those predictions. Specifically, they indicate that in cases in which strong predictions are made (low entropy), these predictions are sufficiently specific to generate a processing penalty on first fixation duration when they are incorrect. To our knowledge this is the first demonstration of a prediction error cost on eye-movement behaviour during natural reading. One question is why this prediction error cost does not also arise in the gaze duration and total reading time data. Our hypothesis is that the prediction error cost in low entropy/high surprisal situations is mitigated in later measures through *integration* processes if the target is semantically similar to the predicted word (see also [15]). To illustrate, while the sentences below would both be expected to generate prediction error on first fixation durations, this prediction error would be mitigated in sentence (a) in later measures due to the semantic similarity between the prediction (denoted in square brackets) and the observed target. Sentences like (b) do not occur in our dataset because the Provo Corpus [29] uses natural language.

(a) The boy was hungry and picked up the shiny red [apple] cherry.
(b) The boy was hungry and picked up the shiny red [apple] button.

#### 3.2.4. Target-prediction similarity

To test this hypothesis, we investigated whether the semantic similarity between the prediction and the target (*target-prediction similarity*) influences fixation durations in cases in which prediction error is expected. To do so, we selected a subset of cases in which the language model had a strong prediction (low entropy) that was ultimately incorrect (high surprisal). To select these cases, we converted the continuous measures of entropy and surprisal to categorical variables with an equal frequency binning (this creates equally distributed categories; arules R package [41]), then selected targets with low entropy and high surprisal values, resulting in a subset of 34 020 observations representing 84 participants × 405 tokens.

**Figure 6.**
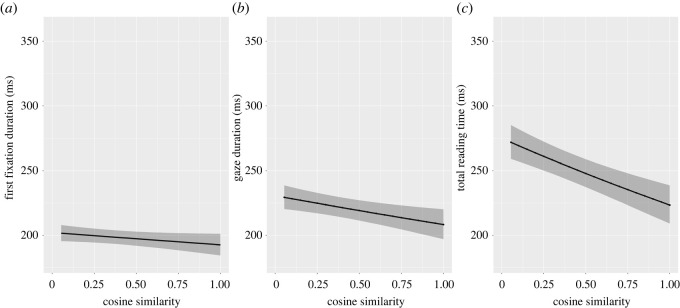
Model estimates of the effect of target-prediction similarity on reading times for 405 tokens (34 020 observations) in which the language model made a strong prediction that was incorrect (low entropy/high surprisal). Target-prediction similarity had a significant effect on human gaze durations and total reading times for these items.

We then analysed whether target-prediction similarity influences fixation durations in this subset of items. In other words, when readers have strong predictions given the preceding context, are they less surprised to see an unexpected target word if it is semantically related to the prediction than when it is unrelated? The effect of target-prediction similarity was analysed with the following formula: *DV ∼ Similarity + Freq + Length + WordNumberInSentence + (1|ParticipantID) + (1|WordID) + (1|TextID)*. Results showed a main effect of target-prediction similarity on gaze duration (β=−0.02, s.e.=0.01, t=−2.94, p=0.003) and total reading time (β=−0.03, s.e.=0.01, t=−5.13, p<0.001). These results suggest that fixation durations are reduced in later reading time measures when there is greater semantic similarity between incorrect predictions and targets (figure 6). There was no statistically significant effect of target-prediction similarity on first fixation duration (β=0.01, s.e.<0.01, t=1.76, p=0.08) or regressions (OR=0.96, s.e.=0.04, z=−1.22, p=0.22).

The fact that target-prediction similarity effects are observed on later measures (gaze duration and total reading time) supports our hypothesis that integration processes mitigate the downstream impact of prediction error. However, our analyses also suggest that these integration processes are not sufficiently rapid to mitigate the impact of prediction error on first fixation durations.

## 4. Discussion

The field of natural language processing has been revolutionized by the development of language models that are sensitive to context [42,43]. These models do not claim to solve natural language processing problems in the same way as people do, and researchers are only just beginning to consider whether and how they can improve understanding of human language and language processing (e.g. [44]). However, use of GPT-2 [30] in this instance has allowed us to make a series of important advances in our understanding of the mechanisms that underpin skilled reading.

These advances arise because GPT-2 allows us to quantify with a high degree of accuracy the prediction distribution *prior* to encountering a target (including for the substantial proportion of words with a cloze probability of zero; figure 2*a*), and to separate this from the degree of surprise *when* a target is reached. Our analyses revealed that GPT-2's top prediction was accurate for 31% of words in the Provo Corpus [29], and that this figure rose to 61% when its top-10 predictions were considered (figure 2*b*,*c*). This performance counters long-standing and influential claims that natural language is too unconstrained for routine prediction to be viable [8]. Moreover, the prediction performance achieved by the model is based on relatively superficial language input: the occurrence of word strings and their co-occurrence with other word strings. One might argue that humans' broader experience of language and their grounded understanding of meaning would permit even better prediction. In short, we believe that an argument against routine use of prediction that invokes the unconstrained nature of language is untenable.

There has been increasing interest in relating language model surprisal to reading times (e.g. [45,46]). However, the distinction that we make between entropy and surprisal in this work is important because it allows us to disentangle prediction from integration processes. Entropy is a measure of what the reader expects to see, and its influence on reading *must* be ascribed to prediction. By contrast, surprisal reflects a reader's reaction to the word that they actually encounter, and its influence on reading may be ascribed to prediction and/or integration [6]. Investigating how both of these metrics map onto events in the eye-movement record has allowed us to make a series of observations that yield new insight into how prediction and integration processes work together across the time course of reading. These observations ultimately lead to a new perspective on how prediction is used to facilitate rapid, skilled reading.

Our results showed that the degree of uncertainty prior to encountering a target (entropy) influences the earliest moments of word recognition before a target is even fixated (figure 3). Entropy influenced the probability of skipping the next word; however, this effect was qualified by an interaction with target surprisal. Higher surprisal targets were less likely to be skipped than lower surprisal targets, but only in higher entropy (higher uncertainty) contexts. These results challenge decades-old thinking about the computations involved in the planning of saccades in reading. Previous research has converged on a view in which analysis of information in the parafovea determines whether a subsequent target will be skipped [40] (see [5] for discussion). Our findings suggest instead that the strength of predictions determines how much information readers sample from the parafovea in planning the next saccade. If readers' predictions are strong, then they act on the basis of those predictions; this strategy is likely to support reading speed, although a substantial proportion of words in our analysis appear to have been skipped based on inaccurate predictions. Our findings suggest that when readers' predictions are not as strong, they sample more information from the parafovea, and hence are less likely to skip higher surprisal targets.

These observations imply that readers may be using predictions dynamically to guide how the eyes interrogate upcoming text. Our findings suggest that it is possible to make accurate next-word predictions in a substantial proportion of cases in natural language (figure 2). Thus, it may be that the routine use of prediction allows readers to adopt a relatively superficial reading strategy, in which deep analysis is reserved for higher uncertainty contexts. If so, this would go some way to explaining the remarkable speed of skilled reading. Our results pertaining to first fixation durations provide further insight into the nature of those predictions. In low uncertainty situations in which readers' predictions are accurate (low entropy/low surprisal), readers enjoy a first fixation benefit (figure 4*a*). However, in low uncertainty situations in which predictions are inaccurate (low entropy/high surprisal), a prediction error cost is observed (figure 4*a*). The observation of this processing cost is important because it provides some of the first evidence that the predictions being made are for specific words (rather than more general semantic or syntactic categories) [5].^2^ Our novel analysis of target-prediction similarity further suggests that this prediction error may be resolved through integration processes operating later in the time course of recognition. Specifically, we observe significant reduction in gaze duration and total reading times when the incorrect prediction is semantically similar to the target; this is likely to be the usual state of affairs in natural language. However, this integration process is not fast enough to mitigate the impact of prediction error on first fixations (figure 6).

The prediction error cost observed arises in situations in which it is possible to make strong next-word predictions. However, there will also be instances in which predictions are weak (high entropy). We have already seen that in these cases, readers' decisions about whether to skip the next word appear to involve greater sampling of information about the identity of that word in the parafovea (as compared to low entropy cases). The sampling of information in the parafovea may help us to understand why the effect of surprisal appears to be attenuated in first fixation durations in higher entropy contexts (figure 4*a*). If readers are sampling enough information from the parafovea to generate an influence of surprisal on the decision to skip the target, then perhaps that sampling is also sufficient to mitigate the effect of surprisal if the eyes land on the target. Further research is necessary to develop this account and to determine why the effect of surprisal in high entropy cases returns in the gaze duration measure.

Our findings give pause to consider how predictive processes may contribute to human mastery of the reading skill. Unlike spoken language, reading is a cultural invention and a learned skill. Yet, literate adults consume linguistic information via reading at speeds that eclipse their engagement with spoken language [1]. Our findings suggest that the language used in text is relatively predictable based on the text statistics available to GPT-2 and that readers routinely use prediction to guide their processing of upcoming words. It may be that literate adults' reading speeds are achieved because deep analysis of individual words is required only infrequently; for example, when uncertainty is unusually high or when predictions are inaccurate. Superficial, prediction-guided reading may be sufficient for understanding in most cases. We use the word *superficial* because the online predictive process that we envisage does not involve deep analysis of the word being encountered. However, we recognize that a reader's ability to engage in this type of predictive processing most likely requires extensive language knowledge and understanding. If the ability to deploy accurate predictions rapidly is a hallmark of skilled reading, then it would be important to understand how the statistical knowledge that permits accurate prediction is acquired, and how this knowledge contributes to the development of reading fluency.

In summary, our work demonstrates that the internal states of a modern language model predict fine-grained aspects of eye-movement behaviour: the decision to land on a word, how long to spend on it, and whether to regress back to it. These models do not strive to mimic human language processing mechanisms. However, they do offer a compelling description of next-word prediction based on statistical information pertaining to the occurrence of words in text. By studying how predictability metrics derived from these models relate to human eye-movement behaviours, we have been able to develop a new perspective on skilled reading that involves coordination of rapid prediction based on statistical information and slower integration based on deeper analysis. It will be important to operationalize and test this account through next-generation computational models of reading, and we believe that language models like GPT-2 may provide a foundation for some of that computational work. Likewise, we believe that understanding how internal model states map onto online reading difficulty opens new opportunities for advances in natural language applications such as readability and summarization. Research at the interface of cognition, neuroscience and natural language processing is beginning to emerge (e.g. [47]) and offers significant opportunity for novel theoretical insight and application.

## Data Availability

The materials, data and analysis scripts for this project can be found at https://osf.io/ypeb2/.

## Footnotes

1 Further analysis revealed that 43% of the low entropy/high surprisal cases were skipped (where these categories were determined by equal frequency binning). These are cases in which there is a strong but inaccurate prediction. This result suggests that a substantial proportion of skips may be based on incorrect predictions.

2 We believe that the insensitivity of the cloze task undermined previous attempts to demonstrate prediction error cost on eye fixations [12] (see the OSF site for this project (https://osf.io/ypeb2/) for further details).
